# Alzheimer’s disease multiple intervention trial (ADMIT): study protocol for a randomized controlled clinical trial

**DOI:** 10.1186/1745-6215-13-92

**Published:** 2012-06-27

**Authors:** Christopher M Callahan, Malaz A Boustani, Arlene A Schmid, Mary G Austrom, Douglas K Miller, Sujuan Gao, Carrie S Morris, Mickey Vogel, Hugh C Hendrie

**Affiliations:** 1 Indiana University Center for Aging Research, 410 West 10th Street, Indianapolis, IN, 46202-3012, USA; 2 Department of Medicine, Indiana University School of Medicine, 714 North Senate Avenue, Indianapolis, IN, 46202-5178, USA; 3 Regenstrief Institute, Inc, 1050 Wishard Boulevard, Indianapolis, IN, 46202-2872, USA; 4 Department of Occupational Therapy, Indiana University School of Rehabilitation Science, 1140 West Michigan Street, Indianapolis, IN, 46202-5199, USA; 5 Roudebush Veterans Affairs Medical Center, 1481 West 10th Street, Indianapolis, IN, 46202-2884, USA; 6 Department of Psychiatry, Indiana University School of Medicine, 714 North Senate Avenue, Indianapolis, IN, 46202-5178, USA; 7 Indiana Alzheimer Disease Center, 635 Barnhill Drive, Indianapolis, IN, 46202-5120, USA; 8 Department of Biostatistics, Indiana University School of Medicine, 714 North Senate Avenue, Indianapolis, IN, 46202-5120, USA; 9 Wishard Health Services, 1001 West 10th Street, Indianapolis, IN, 46202-2879, USA

**Keywords:** Alzheimer’s disease, behavioral interventions, functional decline, primary care

## Abstract

**Background:**

Given the current lack of disease-modifying therapies, it is important to explore new models of longitudinal care for older adults with dementia that focus on improving quality of life and delaying functional decline. In a previous clinical trial, we demonstrated that collaborative care for Alzheimer’s disease reduces patients’ neuropsychiatric symptoms as well as caregiver stress. However, these improvements in quality of life were not associated with delays in subjects’ functional decline.

**Trial design:**

Parallel randomized controlled clinical trial with 1:1 allocation.

**Participants:**

A total of 180 community-dwelling patients aged ≥45 years who are diagnosed with possible or probable Alzheimer’s disease; subjects must also have a caregiver willing to participate in the study and be willing to accept home visits. Subjects and their caregivers are enrolled from the primary care and geriatric medicine practices of an urban public health system serving Indianapolis, Indiana, USA.

**Interventions:**

All patients receive best practices primary care including collaborative care by a dementia care manager over two years; this best practices primary care program represents the local adaptation and implementation of our prior collaborative care intervention in the urban public health system. Intervention patients also receive in-home occupational therapy delivered in twenty-four sessions over two years in addition to best practices primary care. The focus of the occupational therapy intervention is delaying functional decline and helping both subjects and caregivers adapt to functional impairments. The in-home sessions are tailored to the specific needs and goals of each patient-caregiver dyad; these needs are expected to change over the course of the study.

**Objective:**

To determine whether best practices primary care plus home-based occupational therapy delays functional decline among patients with Alzheimer’s disease compared to subjects treated in the control group.

**Outcomes:**

The primary outcome is the Alzheimer’s Disease Cooperative Studies Group Activities of Daily Living Scale; secondary outcome measures are two performance-based measures including the Short Physical Performance Battery and Short Portable Sarcopenia Measure. Outcome assessments for both the caregiver-reported scale and subjects’ physical performance scales are completed in the subject’s home.

**Randomization:**

Eligible patient-care giver dyads will be stratified by clinic type and block randomized with a computer developed randomization scheme using a 1:1 allocation ratio.

**Blinding:**

Single blinded. Research assistants completing the outcome assessments were blinded to the subjects’ treatment group.

**Trial status:**

Ongoing

**ClinicalTrial.Gov identifier:**

NCT01314950; date of completed registration 10 March 2011; date first patient randomized 9 March 2011

## Background

Dementia is a growing public health problem with the prevalence varying from 3 to 11% among people aged 65 years and over [1]. Dementia leads to a high burden of suffering for patients, families, and society with an annual estimated cost of $100 billion in the US [2,3]. There were an estimated 7 million cases of dementia in the US in 2000 and this number may grow to 18.5 million by the year 2050 [4]. Sloane and colleagues estimated that the number of Alzheimer’s disease (AD) cases in the US would rise from 2.7 million in 2005 to 10 million in 2050 if there are no important advances in current treatment strategies [5]. If treatments were discovered that both delayed disease onset and slowed disease progression, the number of cases would still rise to more than 6 million [5]. Even under optimistic scenarios of improved treatment, we must prepare to care for a growing population of older adults with AD. Thus, research efforts must focus on care in addition to cure.

Most older adults with AD receive their medical care in primary care settings yet most primary care physicians care for fewer than two dozen older adults with AD [6,7]. Researchers and policy makers consistently document suboptimal quality and poor outcomes among older adults receiving the usual care of generalist physicians [8-11]. There have been three general responses to this persistent quality problem in primary care [12,13]. The first has been to improve the knowledge, skills, attitudes, and behavior of primary care physicians. The second approach has been to add resources into the primary care setting. The third approach has been to virtually expand primary care through information technology, facilitated access to care managers or specialists, and improved coordination of care such as patient-centered medical home approaches [14]. A non-primary care approach has been to simply move high-need patients to another setting such as a specialty dementia clinic. These interventions are not mutually exclusive and each provides certain benefits.

In 2006, we reported the results of a randomized controlled clinical trial testing the effectiveness of collaborative care compared with augmented usual care among primary care patients with AD [15]. The primary care-based collaborative care intervention resulted in statistically significant improvement in the quality of care and in behavioral and psychological symptoms for patients and their caregivers. The improvements we reported in this trial on the intervention subjects’ neuropsychiatric inventory scores were among the highest reported in the literature at that time [15]. However, guideline-level care did not slow the rate of patients’ functional decline compared with augmented usual care. Notably, functional decline among older adults with AD is often due to comorbid conditions in addition to the AD [16]. Also, functional decline, as compared with behavioral symptoms, may be a stronger predictor of subsequent institutionalization among older adults with AD [17]. Several short-term studies focusing specifically on functional decline among patients with AD and related dementias have shown the potential to slow functional decline through home-based interventions [18-20]. The current study builds on our past work and those of others by integrating home-based occupational therapy interventions with our primary care-based collaborative care intervention to delay functional decline.

The specific aim of this study is to conduct a two-year randomized, controlled clinical trial to delay functional decline among older adults with AD by comparing a control group receiving best practice primary care with an intervention group receiving best practice primary care plus a home-based occupational therapy intervention. We will test the primary hypothesis that subjects with AD in the intervention group will have better function at two years compared with the best practice primary care control group, as measured by the Alzheimer’s Disease Cooperative Studies (ADCS) Group Activities of Daily Living (ADL) Inventory. Because this inventory is self-reported by the caregiver, we will also assess patient function using two physical performance tests. ‘Best practice primary care’ is not equivalent to the usual care of most primary care practices. In prior work in primary care settings, we have demonstrated the limitations of usual care for patients with dementia [7,9]. Thus, we are seeking to determine if our combined intervention can slow the rate of functional decline when compared with best practice based on our prior approach. We also hypothesize that tailored occupational therapy will bring incremental benefits in behavioral outcomes over and above those demonstrated in our prior intervention.

## Methods/design

### Design

**Figure 1:**
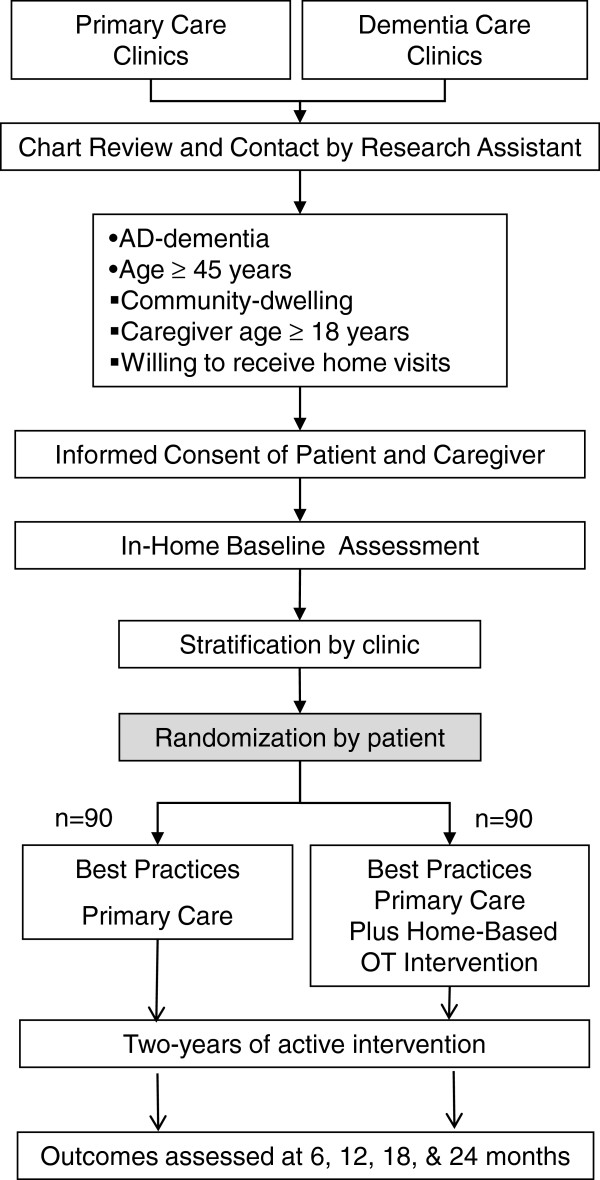
Study design.

The overall study design is shown in Figure 1. This is a randomized single blind controlled clinical trial with a parallel design and a 1:1 allocation ratio. We will enroll a total of 180 care-recipient and caregiver dyads with all care-recipients meeting diagnostic criteria for possible or probable AD. The study was approved by the Indiana University - Purdue University Indianapolis Institutional Review Board (IRB number 0907–82). Study safety is also monitored by an Independent Data Safety Monitor who is a single individual approved by the funding agency and selected from an external institution. The role of the Independent Data Safety Monitor is to review study progress, data quality, and unanticipated adverse events.

### Setting

The study site is Wishard Health Services, an urban public health system serving medically indigent patients in Indianapolis. Wishard Health Services includes a 350-bed hospital and a network of eight primary care centers in Indianapolis. It also includes a Senior Care program staffed by faculty in an academic geriatric medicine program that includes services such as an Acute Care for Elders Unit, a physician house calls program, and specialty geriatric ambulatory care services provided in the Center for Senior Health [21]. Geriatric ambulatory care services include the Healthy Aging Brain Center (HABC) [22] and the Aging Brain Care-Medical Home (ABC-MedHome) [23]. Both of the aging brain clinic sites are local adaptations and implementation of the collaborative care program tested in a prior clinical trial [15]. Both the HABC and the ABC-MedHome support primary care providers through collaborative care, however, HABC provides this care in a dementia care clinic while the ABC-MedHome provides this care in the home setting. Some patients may be seen in both programs.

### Type of participants

Patients are recruited from the HABC, the Center for Senior Health, and the primary care practices affiliated with Wishard Health Services that are served by the ABC-MedHome. The older adult population cared for at Wishard included approximately 7,500 adults aged 65 years and older in 2009. Nearly all of these patients have at least one visit to their primary care physician every two years. Based on our prior studies, we have a wealth of data on this population. Briefly, approximately 68% are women, approximately 64% are African-American, approximately 40% had ≤8 years of education, and approximately 50% are dually eligible for both Medicare and Medicaid. The prevalence of dementia is 6% based on prior formal screening programs in these primary care clinics [7]. Chronic medical illnesses are common including hypertension (63%), arthritis (30%), diabetes mellitus (25%), chronic obstructive lung disease (18%), coronary artery disease (15%), severe obesity (14%), cancer (10%), and congestive heart failure (8%), among others [7,9,16,24,25].

Patients are eligible if they are aged 45 years and older and have a diagnosis of possible or probable AD, including those patients who may have a mixed dementia. Original eligibility criteria included age 65 years and older, but this age limit was reduced based on interest in the study by younger potential subjects at the outset of the trial. Eligibility criteria also include: receiving primary care at Wishard Health Services, community-dwelling, English-speaking, a caregiver willing to participate in the study, and willing to receive home visits. Caregiver eligibility criteria includes age 18 years or older, English-speaking, and regular access to a telephone. For patients meeting eligibility criteria, research personnel assigned to each clinical site obtain informed consent from the patient and the family caregiver. For patients unable to provide informed consent due to their level of cognitive impairment, we seek to obtain assent from the patient as well as informed consent from their legally authorized representative. Prior to randomization and within two weeks of enrollment, patients and their caregivers complete an in-home baseline assessment by a professional research assistant. This research assistant is blinded to the subjects’ ultimate randomization status and is not involved in delivering the intervention. Enrollment will take place over 2.5 years (30 months) with an enrollment target of 180 subjects and their caregivers.

### Description of the control condition

All enrolled patients and their caregivers (both best practice primary care and intervention patients and regardless of their enrollment site) will receive collaborative care for dementia through the ABC-MedHome with or without HABC. All subjects will be provided each of the components of best practice primary care as listed in the overview below:

written materials and face-to-face counseling about their diagnosis

written materials describing local community resources, including access to the local chapter of the Alzheimer’s Association

written consultation note to the patient’s primary care physicians communicating the results of the diagnostic assessment and the patient’s participation in the study

collaborative care management including:

medical co-management within the ABC-MedHome in collaboration with the patient’s primary care physician and the geriatrician, dementia-care advanced practice nurse and social worker affiliated with the ABC-MedHome, including:

treatment with cholinesterase inhibitors (or memantine) unless contraindicated;

education on communication skills; caregiver coping skills; and legal or financial advice;

a caregiver guide provided by the Alzheimer's Association;

enrollment in the local Alzheimer’s Associations safe return program;

treatment of behavioral disturbances based on established protocols that first emphasize non-pharmacological approaches;

longitudinal telephone-based support

tracking of patient outcomes including behavioral symptoms of dementia

access to a monthly support group for caregivers within the targeted primary care clinics.

We consider all of the above ‘best practice primary care’ because they encompass the collaborative care intervention tested in our prior clinical trial [15]. Because these interventions are not typical of usual care, we recognized that the true impact of the intervention on behavioral outcomes may appear diminished in our results. However, we are trying to determine if the addition of home-based occupational therapy is able to forestall functional decline that the prior intervention did not achieve. Best practice primary care will be provided for both experimental groups for 24 months. This care is led by the patient’s primary care physician and a geriatric nurse practitioner who serves as the dementia care manager. One care manager can provide collaborative care to about 80 to 100 patients in steady state. Caregivers and patients are seen by the dementia care manager at home, in the primary care clinic, or in other clinical sites, but home visits are the most common. Early contacts are dominated by face-to-face meetings and later contacts tend to be dominated by telephone contacts, depending on the clinical course of the patient and caregiver.

At each contact with the care manager, caregivers complete the Health Aging Brain Care (HABC) Monitor. The HABC Monitor includes 31 items covering the four clinical relevant domains of dementia symptoms; cognition, functional deficit, behavioral/psychological, and caregiver burden. Each item has four categories of responses that use the frequency of the target problem in the past two weeks and has a similar anchor. Total scores on the HABC Monitor vary from 0 to 124 with higher scores indicating greater patient symptomatology and caregiver stress. The HABC Monitor demonstrated good internal consistency (0.73 to 0.92); test-retest reliability; construct validity indicated by correlations with the caregiver-reported Neuropsychiatric Inventory (NPI) total score and NPI caregiver distress score; responsiveness to three-month change compared to NPI ‘reliable change’ groups; and known-groups validity indicated by significant separation of Mini Mental Status Examination (MMSE) severity groups and clinical diagnostic groups. (Description and psychometric properties available at http://www.indydiscoverynetwork.org//HealthyAgingBrainCareMonitor.html.) Based on the caregiver's reports of the patient’s current symptoms, individualized recommendations are made regarding how to manage a patient's behavioral symptoms [26]. Items reported by the caregiver dictate activation of specific behavioral intervention protocols by the care manager. There are ten protocols including personal care, repetitive behavior, mobility, sleep disturbances, depression, agitation or aggression, delusions or hallucinations, the caregiver's physical health, driving safety, nutrition, and delirium. Each of these protocols focuses first on non-pharmacological interventions. If the non-pharmacological approach does not help, the care manager collaborates with the primary care physician to consider the protocol-based drug therapy for those behavioral problems that could be amenable to pharmacologic intervention (for example, depression).

The primary care physician and the care manager have access to support and expert back-up from providers in the HABC. This support can occur either through telephone consultation or face-to-face meetings with the care manager. The care manager and primary care physician can also utilize referral to specialty care. The care manager is supported by a Web-based longitudinal tracking system that manages the schedule for patient contacts, tracks the patient's progress and current treatments, and provides a mechanism for communicating the patient's and caregiver's current clinical status to the entire care team. Patients and their caregivers will also be offered access to support groups affiliated with their clinic.

### Description of the intervention condition

The intervention group receives all of the components of best practice primary care described above. In addition, this group receives a home-based intervention designed to slow functional decline. The main framework of the intervention is based on general occupational therapy principles and prior published research [18-20,27]. Our main goal is to support and augment self-care functioning capability of the patient as identified by goals established in negotiation with the patient and caregiver. Thus, the focus is to lessen the impact of the dementing illness and attendant comorbid conditions to minimize: (a) decline in the patients’ functioning and physical performance abilities; (b) caregivers’ stress, and (c) likelihood that the patient will need formal services. The intervention takes place in the patient’s home and is led by an occupational therapist. The occupational therapist completes an initial evaluation to develop a formal care plan that is tailored to the needs of the individual patient-caregiver dyads. Specific therapeutic interventions will be tailored for each individual or caregiver dyad based on the standardized evaluation and patient and caregiver goal identification. Thus, we expect that each dyad’s specific constellations of training and support will vary. The content of the occupational therapy initial assessment includes: Caregiver Assessment of Management Problems (CAMP) [28]; Fear of Falling (FoF); Berg Balance Scale [29]; Functional Independence Measure (FIM) [30]; Allen’s Cognitive Level Screen (ACLS) [31]; and the Mini Nutritional Assessment (MNA) [32].

An overview of the intervention is summarized in Table 1. The five assessment tools described above are completed at the beginning of each of the three home-based cycles to tailor the home-based component for individual dyads at each cycle. The therapist may also repeat this assessment at any time that they adjudge a major change in the patient’s clinical status. Thus, at minimum, this evaluation is done at baseline, 16 weeks from baseline, and 48 weeks from baseline (three times) and at minimum, three care plans will be completed.

**Table 1:** Overview of occupational therapy (OT) in-home intervention

**Time**	**OT intervention with care-recipient**	**OT intervention with caregiver**
**Week One**	Initial Evaluation, CAMP, FoF, Berg, FIM, MNA, and ACLS	Identify goals with patient and caregiver Identify patient and caregiver abilities Coordinate plans with dementia care manager
**Week Two**	Discuss care plan with patient and caregiver Set activity goals	Discuss care plan with patient and caregiver**CG Goal:** Decrease risk of caregiver injury
	**New Task:** Identify meaningful activity	
**Week Four**	Review care plan with patient and caregiver	**New Task:** Train caregiver to safely assist in
	**New Tasks:** ADL training/Transfer training	activities of daily living and transfer training
**Week Six**	**New Tasks:** Begin strength training and exercise and	**New Tasks:** Begin strength training and exercise
	provide safety information	Issue safety information
	Focus on meaningful activity	Focus on meaningful activity
**Week Eight**	Review ADL and exercise training	Review ADL training, cognitive training, and
	Address cognitive training	exercise programming
	Time for meaningful activity	**New Task:** Medication management
	**New Task:** Medication management	
**Week Ten**	Review ADL training and exercise programming	Review ADL training and exercise programming
	Address meaningful activity	**New Task:** Work with caregiver to identify IADL
	**New Task:** IADL training	needs
**Week Twelve**	Review ADL training and exercise programming	Review ADL training and exercise programming
	Time for meaningful activity	
	Address cognitive training	
	**New Task:** Identify one way to increase social participation	
**Week Sixteen**	Review ADL training and exercise programming	Review ADL training and exercise programming
	Review IADL training	Review IADL training
	Review safety information	Review safety information
	Time for meaningful activity	

ADL, activities of daily living; ACLS, Allen’s Cognitive Level Screen; Berg, Berg Balance Scale; CAMP, Caregiver Assessment of Management Problems; FIM, Functional Independence Measure; FoF, Fear of Falling; IADL, Instrumental Activities of Daily Living; MNA, Mini Nutritional Assessment.

There will be three cycles of the home-based intervention over two years. In the first cycle, there will be eight 90-minute sessions delivered approximately every other week over 16 weeks with a telephone call in intervening weeks. A new task is introduced with each visit based on a mutually agreed upon care plan. Additional phone contacts are allowed to assist with problem solving and address interval problems. At the end of cycle one (16 weeks), the occupational therapist will repeat the standardized assessment, construct a newly tailored care plan, and then complete another eight-session cycle, but in the second cycle the eight home visits will be spaced by four weeks and therefore take place over 32 weeks. In the second year, (the third cycle of the home-based component), the protocol will also begin with the same assessment and a newly tailored protocol. In the third cycle, the eight home visits will take place over one year. Thus, each patient will receive up to twenty-four 90-minute homes visits over two years but the visits are more closely clustered in the first year.

### Outcome measures

Outcomes measures are completed in the home by a team of two research assistants who are blinded to the dyad’s randomization status. One of the research assistants focuses on the interview of the caregiver and the second research assistant focuses on the performance-based measures of the care-recipient. Outcome assessments are completed at baseline six, twelve, eighteen, and twenty-four months.

#### Primary outcome measure

The primary outcome measure is the ADCS Group Activities of Daily Living Inventory. This is a 23-item inventory developed by the ADCS Group that is administered to the patient’s caregiver by a trained interviewer. The caregiver is asked to focus on the patient’s performance over the past month. Notably, the caregiver reports on what the patient actually did rather than an assessment of what the patient might be able to do. Thus, the Inventory focuses on observed actions. The items were chosen from among 45 items rating activities of daily living reported in the literature. The instrument assesses the traditional basic activities of daily living as well as variations on instrumental activities of daily living and a number of more complex and explicit self-care tasks [33]. Scores vary from 0 to 75 with higher scores indicating greater levels of function.

#### Secondary outcome measures

The Neuropsychiatric Inventory (NPI) has been adopted by the ADCS Group to obtain information on the presence of psychopathology in behavioral areas including delusions, apathy, hallucinations, disinhibition, agitation, depression, aberrant motor behavior, anxiety, night-time behavior, and euphoria. Scores vary from 0 to 144 with higher scores representing worse symptoms. The Inventory is interviewer administered to a caregiver. The NPI can be used to assess changes in the patient’s behavior over the past month or other specified time intervals. If the caregiver reports the presence of psychopathology, there are follow-up questions to assess frequency, severity, and the level of caregiver distress due to the behavior. Thus, the instrument is specifically designed to also measure caregiver distress (possible scores vary from 0 to 60). The administration time is about 20 minutes. The test has excellent reliability and validity [34-36].

The Short Physical Performance Battery (SPPB) is a standardized measure of lower extremity physical performance that includes walking, balance, and power tasks, and has been used in a broad range of epidemiological studies of aging [37-40]. This scale has proven reliable and valid for predicting disability, nursing home placement, hospital admission, and mortality [40-44]. The SPPB score is based on timed measures of standing balance, walking speed, and repeated chair rises. Scores vary from 0 to 12 with higher scores indicating better function.

The Short Portable Sarcopenia Measure (SPSM) was conceptualized as a measure of sarcopenia that combines muscle quantity and function [45]. The SPSM can be used to follow change in muscle status over time with each person as his or her own control. The scale is based on timed chair rises, lean mass, and grip strength divided by height. Scores vary from 0 to 18 with higher scores indicating better function.

Additional outcome and process of care data collected for this study are listed in Tables 2 and 3. These data are collected at baseline, and then at 6, 12, 18, and 24 months from the time of the baseline date.

**Table 2:** Additional outcome data

**Completed with care-recipient**	**Completed with caregiver**
Mini Mental State Examination (MMSE) [46]	ADCS Resource Use Instrument [47]
Word List Learning Test [48]	Patient Health Questionnaire-9 (PHQ-9) [49]
Mini Nutritional Assessment (MNA) [32]	Generalized Anxiety Disorder-7 (GAD-7) Scale [50]
Blood pressure, height, and weight	Satisfaction with the care
body composition (by bioelectrical impedance analysis)	Adverse Event Checklist

**Table 3:** Process of care data

***Provider contacts***
Dementia care specialists (APN, RN, or MSW)
Number of home visits, mean
Number of clinic visits, mean
Number of telephone contacts, mean
Occupational therapist
Number of home visits, mean
Number of clinic visits, mean
Number of telephone contacts, mean
Total contacts
Other rehabilitation therapist visits
Physicians
Number of primary care MD visits, mean
Number of specialty dementia MD visits, mean
Number of other specialty MD visits, mean
*Dementia Care*
Pharmacologic management
Number (%) receiving anti-dementia drug
Number (%) receiving antipsychotic drug
Number (%) receiving antidepressant
Number (%) receiving sedative-hypnotic
Anticholinergic burden
Percentage on definite anticholinergic medications
Percentage on cholinesterase inhibitor and anticholinergic medications
Percentage with anticholinergic medication discontinued
Non-pharmacologic management
Family conference to reveal diagnosis
Written educational materials
Contact information for Alzheimer’s Association
Number of behavioral protocols activated
Number receiving dietary supplement

APN, advanced practice nurse; MD, medical doctor or doctor of medicine or physician; MSW, master of social work; RN, registered nurse.

### Sample size

The targeted sample size is 180 patients. The study is designed to have at least 80% power for testing the following hypothesis based on two-tailed tests at 5% significance level: subjects with AD in the intervention group will have improved function at two years compared with the best practice primary care control group. The primary outcome is the ADCS Group ADL Inventory measured at 24 months. The expected effect size for the primary outcome of ADL Inventory in Table 4 is based on clinically important differences that can be realistically expected from published studies [18,20,27]. Since baseline values will always be included as covariates in all between-group comparisons, the residual variance used in power calculation is equal to the population within-group variance multiplied by (1-correlation^2^), with correlations estimated from our prior clinical trial [15]. We estimated that we will have 82% power to detect the between-group difference in ADL Inventory at 24 months. The software package nQuery was used to conduct the sample size calculation [51].

**Table 4:** Expected effect size

	**Assumed true intervention effect**	**Expected between-group difference**	**Pooled within-group SD (σ)**	**Correlation with baseline value**	**Statistical power(90 subjects/group)**
Activities of Daily Living Inventory	0.23σ to 0.31σ	0.23σ (mean of 0.20σ and 0.26σ)	17.4	0.85	82%

### Randomization

Randomization will be conducted at the patient level stratified by clinic (Healthy Aging Brain Center or all other ambulatory care clinics). The statistical software SAS (SAS Institute Inc. 2008. SAS/STAT 9.2 User’s Guide. SAS Institute Inc., Cary, NC, USA) was used to generate the randomization scheme. Sequentially numbered sealed envelopes containing the randomization assignment for patient for each of the two clinic types were prepared by the study statistician. Actual randomization results will be compared to pre-planned randomization schedule to ensure randomization integrity.

### Data management

Outcome assessment and OT measures are entered into a REDCap (Research Electronic Data Capture) database, an electronic data capture tool hosted at Indiana University Clinical Translational Science Institute [52]. REDCap is a secure, Web-based application designed to support data capture for research studies, providing validated data entry, audit for tracking data manipulation and export procedures, automated export procedures and procedures for importing data from external sources.

### Primary analysis

The efficacy of the clinical trial will be tested on the primary outcome of ADL Inventory measured at 24 months. Analysis of covariance (ANCOVA) will be used for testing the effect of the intervention while controlling for the baseline ADL Inventory and randomization stratum. We will perform an intention-to-treat analysis on all patients who have the ADL Inventory measured at baseline and at least one other value. Since only those subjects who complete the 24-month assessment will have the primary outcomes measured, multiple imputation will be used in cases of missing data at 24 months. Multivariate normal (MVN) models will be used as the imputation models using patients’ characteristics, baseline and post baseline outcome variables to impute missing ADL values, as previous research indicated the MVN method performed well when compared to the completed cases only analysis or the last observation carried forward technique in simulation studies [53]. These imputed outcomes will then be used in the primary efficacy analyses according to established guidelines [54]. ANCOVA models will also be used to test the secondary hypothesis that the combined intervention will improve the subject’s performance on the two physical performance scales and both patients’ behavioral symptoms and caregiver stress, both of which are captured by the NPI.

## Discussion

The multifaceted intervention tested in this trial builds upon past research in three ways. First, we are integrating a comprehensive set of biopsychosocial care management recommendations for older adults with dementia and their caregivers. These recommendations encompass not only a complex set of medical management options, but also a team-based approach that includes nursing, social work, and rehabilitation sciences care strategies. Second, the intervention is substantially longer than the typical Alzheimer’s disease intervention reported in the literature. Both control and intervention patients will receive care management over a two-year period. Third, we have moved the bulk of this intervention, including the medical management, into the patient’s home to overcome some of the structural limitations of primary care. One of the most important limitations in the primary care environment is space. The study is ongoing and has now completed 12 months of enrollment with 75 patient-caregiver dyads enrolled as of February 2012. There have been no serious adverse events to date and both caregivers and care-recipients have been receptive to the interventions.

## Trial status

Ongoing.

## Abbreviations

ABC-MedHome, Aging Brain Care-Medical Home; ACLS, Allen’s Cognitive Level Screen; ADL, Activities of Daily Living; AD, Alzheimer’s disease; ADMIT, Alzheimer’s Disease Multiple Intervention Trial; ADCS, Alzheimer’s Disease Cooperative Studies; ANCOVA, analysis of covariance; APN, advanced practice nurse; Berg, Berg Balance Scale; CAMP, Caregiver Assessment of Management Problems; FIM, Functional Independence Measure; FoF, Fear of Falling; GAD, Generalized Anxiety Disorder; HABC, Healthy Aging Brain Center; IADL, Instrumental Activities of Daily Living; IRB, Institutional Review Board; MD, medical doctor or doctor of medicine or physician; MMSE, Mini Mental Status Examination; MNA, Mini Nutritional Assessment; MSW, master of social work; MVN, multivariate normal; NIA, National Institute on Aging; NPI, Neuropsychiatric Inventory; OT, occupational therapist; PHQ, patient health questionnaire; REDCap, Research Electronic Data Capture; RN, registered nurse; SPPB, Short Physical Performance Battery; SPSM, Short Portable Sarcopenia Measure.

## Competing interests

The authors declare that they have no competing interests.

## Authors’ contributions

CC, MB, AS, MA, DK, SG, and HH made contributions to the conception and design of the study. CM and MV made contributions to the acquisition of data. All authors participated in drafting the manuscript and revising it critically for important intellectual content. All authors have given final approval of the version to be published.

## Funding

National Institute on Aging (R01 AG034946).
